# Dietary *Astragalus* Polysaccharides Can Improve the Immune Capacity and Reproductive Performance of the Lined Seahorse (*Hippocampus erectus*)

**DOI:** 10.3390/biology14070767

**Published:** 2025-06-25

**Authors:** Siping Li, Xin Liu, Tingting Lin, Yuanhao Ren, Dong Zhang, Keji Jiang

**Affiliations:** 1 Key Laboratory of Inland Saline-Alkaline Aquaculture, Ministry of Agriculture and Rural Affairs, East China Sea Fisheries Research Institute, Chinese Academy of Fishery Sciences, Shanghai 200090, China; lisiping@ecsf.ac.cn (S.L.); liux@ecsf.ac.cn (X.L.); renyh@ecsf.ac.cn (Y.R.); zhangdong@ecsf.ac.cn (D.Z.); jiangkj@ecsf.ac.cn (K.J.); 2 Wenchang Innovation Research Center, Fengjiawan Modern Fishery Industry Park, Wenchang 571300, China; 3 Key Laboratory of Tropical Marine Ecosystem and Bioresource, Ministry of Natural Resources of China, Beihai 536000, China

**Keywords:** *Astragalus* polysaccharides, immune capacity, juveniles and broodstocks, reproductive performance, seahorse *Hippocampus erectus*

## Abstract

**Simple Summary:**

In aquaculture, due to the high stocking density, undiversified food, and monotonous environment, the decline in the immune function of cultured animals is a common issue. Therefore, some immunostimulants are usually used to improve the immunity and disease resistance of cultured animals. *Astragalus* polysaccharides (APSs) have been used as immunostimulants in aquaculture for more than 20 years, and their immune-promoting effects have been well-verified in a large number of cultured animals. Seahorses are widely cultivated in China, and recently also face the issue of decline in immune capacity. Up until now, there has been no report of APS application in this animal. In order to test whether APSs would also have immune-promoting effects on seahorses, we conducted several investigations in this study. The results showed that dietary APSs could significantly improve the growth, survivorship, plasma immune levels, and intestinal microbiota diversity of seahorse juveniles as well as the reproductive performance of seahorse broodstocks. These results are valuable for the healthy breeding of seahorses.

**Abstract:**

Seahorse (*Hippocampus* spp.) is popular in the markets of traditional Chinese medicine, aquarium, and curio. In order to protect wild stocks and still meet the market demand, China attempted the large-scale cultivation of seahorses in the early 21st century and achieved it in the 2010s. However, in recent years, two new issues have gradually emerged in Chinese seahorse cultivation. One is that the juveniles are prone to disease during diet conversion, and the other is that the reproductive performance of broodstocks is significantly reduced. With the aim to provide some measures that can alleviate these issues, in the present study, we used lined seahorse (*Hippocampus erectus*, a species widely cultured in China) as the experimental subject and *Astragalus* polysaccharides (APSs) as the immunostimulant to test whether APSs could improve the immune-health status and reproductive performance of seahorses. The measured indices for reproductive performance included ovarian lipid content, assessment time required before mating for paired male and female seahorses, mating success rate, brood size, and newborn body height. The results showed that for juveniles during diet conversion, their body weight, survival rate, plasma immunocytokine contents (interleukin-2, interferon-α, and immunoglobulin M), and alpha diversity indices (Simpson and Pielou’s-e) of the intestinal microbiota were significantly higher than those of the control group after dietary APSs. For broodstocks, compared with the control group, the expression of lipid substances in the ovary was significantly upregulated, the assessment time was significantly shortened, and the body height of their newborns was significantly increased in the APS group. These results demonstrate that APSs could indeed improve the immune-health status and reproductive performance of seahorses, providing guidance for addressing existing issues in seahorse cultivation.

## 1. Introduction

Seahorse (*Hippocampus* spp.) is very popular in traditional Chinese medicine, aquarium, and curio markets. The growing global trade of seahorses has exerted a great pressure on wild stocks, and all species of this genus have been listed in Appendix A of CITES since 2004 [1]. In China, seahorses are mainly used as a traditional Chinese medicine and are in wide demand on the market [2,3]. In order to protect wild seahorses and still meet the strong market demand, China has increased its attempts at the artificial cultivation of seahorses since the early 21st century [4,5,6], and has successfully achieved the large-scale cultivation of some species such as the lined seahorse *H. erectus* [7,8] and the big-belly seahorse *H. abdominalis* [9,10]. The successful cultivation of seahorses not only brings considerable economic benefits to the enterprises engaged in aquaculture or traditional Chinese medicine, but also reduces the dependence on wild seahorses and protects them.

However, in recent years, there have been some new issues in seahorse cultivation, among which two are the most prominent. The first is that seahorse juveniles are prone to disease during diet conversion [8,11]. Seahorse juveniles after birth only feed on live food (e.g., larvae and adults of copepods and cladocerans) for the first two and a half months and start feeding on frozen food (e.g., *Artemia*, *Mysis* and *Acetes*) after two and a half months (their body height reaches at approximately 7.5 cm at this time) [12]. After 10–15 days of consuming frozen food, they begin to show symptoms of disease. The symptoms manifest as diseased seahorses floating on the water surface, weak swimming, inactive feeding, inability to hold on the holdfasts, abdominal swelling, and anal protrusion. After the onset of symptoms, the vast majority of seahorses die within 7 days, with a total mortality rate of up to 60%. The second is that the reproductive performance of seahorse broodstocks has significantly declined compared with the previous level. Specifically, the time required for male and female seahorses from meeting to first mating success is extended (this period is a process of mutual familiarity and assessment), and the continuous breeding frequency of pair-bonded male and female seahorses is also reduced. Even worse, after two or three pregnancies, the brood pouches of some male seahorses begin to contract and harden, even filling with gas that cannot be expelled, leading them to be unable to receive female eggs and temporarily stop breeding. The decline in reproductive performance greatly increases the management and maintenance cost of seahorse broodstocks for aquaculture enterprises.

Regarding the above two issues, some researchers have conducted relevant studies. Tian et al. [11] believe that the reason for the first issue may be the intestinal microbiota dysbiosis of seahorse juveniles caused by frozen food, thereby leading to diseases. Therefore, they suggest supplementing the diet with *Enterococcus faecium* (a probiotic), which can improve the survival rate and intestinal health of seahorse juveniles during diet conversion. Tang et al. [13] and Xue et al. [14] hold that the reason for the second issue may be related to the increase in pollutants in the marine environment. Through toxicological tests, they demonstrated that some marine pollutants (e.g., tributyltin, benzo[a]pyrene) do indeed have an inhibitory effect on the reproductive performance of seahorses. In addition to the possible reasons above-mentioned, we suppose that the decline in seahorse immunity may also be one. Therefore, an improvement in seahorse immunity by adding immunostimulants may help alleviate these two issues.

*Astragalus* polysaccharides (APSs) have been widely used as immunostimulants in aquaculture for more than 20 years. Their beneficial effects, such as the promotion of growth and digestion, enhancement of immune response, antioxidant capacity, and bacterial resistance, and improvement of intestinal homeostasis, have been well-verified in a large number of aquaculture animals such as large yellow croaker (*Larimichthys crocea*) [15], Asian seabass (*Lates calcarifer*) [16], giant freshwater prawn (*Macrobrachium rosenbergii*) [17], Nile tilapia (*Oreochromis niloticus*) [18], turbot (*Scophthalmus maximus* L.) [19], crucian carp (*Carassius auratus*) [20] and rainbow trout (*Oncorhynchus mykiss*) [21]. However, so far, there has been no verification in seahorses. The lined seahorse (*H. erectus*) is naturally distributed in Nova Scotia along the western Atlantic coast through the Gulf of Mexico and Caribbean to Venezuela [22]. Due to its large size, fast growth, diverse body color, and high temperature resistance, it was introduced into China for captive breeding in 2009 and successfully commercialized in about 2015 [6,23]. The lined seahorse is widely cultivated in southern China, such as in Hainan, Guangdong, Guangxi and Fujian, and the annual output (dried specimen) has exceeded 10 tons since 2020.

In the present study, we used the lined seahorse as the experimental subject and employed APSs to test whether it helped improve the immune-health status of juveniles during diet conversion and the reproductive performance of broodstocks during breeding. The measured indices for immune-health status included growth, survival rate, plasma immunocytokine contents (interleukin-2 (IL-2), interferon-α (IFN-α), and immunoglobulin M (Ig M)), and composition and diversity of intestinal microbiota. IL-2, IFN-α, and Ig M are widely reported to be three immunocytokines closely related to the immune status of the host, and their levels largely reflect the host’s immune capacity [24,25]. The intestinal microbiota has been well-demonstrated to play an important role in maintaining the host’s intestinal health and metabolic homeostasis [26]. The measured indices for reproductive performance included female ovarian lipid content, assessment time required before mating for paired male and female seahorses, mating success rate, brood size, and newborn body height. Ovarian lipids are the most important energy source for embryonic development and are an important indicator of female reproductive investment [27]. Paired male and female seahorses from their first encounter to successful mating need a period of time for mutual familiarity and assessment, and usually a longer assessment time is required between pairs with poor health status. The aim of the present study was to provide potential measures to the issues that exist in seahorse cultivation.

## 2. Materials and Methods

### 2.1. Experimental Seahorses and Astragalus Polysaccharides

The present study was carried out at the Seahorse Research Center of the East China Sea Fisheries Research Institute, Qionghai City, Hainan Province, China. The Center has hundreds of thousands of artificially cultivated lined seahorses of various sizes and several sets of standardized aquaculture facilities. These seahorses were fed live copepods for the first two and a half months after birth, and then switched to feeding frozen *Mysis*.

The gender-undifferentiated juvenile seahorses with a body height of approximately 7.5 cm who had not yet switched to feeding frozen *Mysis* were selected as the experimental seahorses for Experiment 1, and the male and female seahorses near sexual maturity (with a body height of approximately 11 cm) who had been feeding on frozen *Mysis* for a period of time were selected as the experimental seahorses for Experiment 2. These experimental seahorses were stored in their respective holding tanks.

*Astragalus* polysaccharides (APSs, Henghuibio^TM^) were purchased from Beijing Rui Da Heng Hui Science & Technology Development Co., Ltd. (Beijing, China), and the purity was over 90% (Product No. HD-6198-25g).

### 2.2. Administration Route of APSs

In numerous aquaculture applications, the administration route of APSs is mostly through adding polysaccharides to the basal diet formula, then drying and pelleting them into formulated feed for feeding [15,16,17]. However, for seahorse cultivation, the current food used is mainly fresh or frozen natural feed, and there is no formulated feed as yet. Therefore, the route of the integration of APSs into the formulated feed is not workable in seahorses. As for the route of simply mixing APSs with frozen food, it may not be very effective. On the one hand, the taste of frozen food may be altered by APSs, thereby affecting the feeding of seahorses. On the other hand, the APSs adhered to the surface of frozen food is easily diluted by seawater, resulting in a limited intake of APSs by seahorses. Therefore, in the present study, we adopted the route of using *Artemia* as the polysaccharide carrier [28], that is, live *Artemia* were soaked in the APS suspension for a period of time, and when the APSs in their bodies was enriched to a certain level, they were then fed to the seahorses.

In order to determine the APS concentration used for soaking *Artemia* and the APS content inside the *Artemia* after soaking, we conducted a preparatory experiment. In this experiment, five different concentrations (i.e., 0, 2, 4, 8, 16% (*w*/*v*)) of APS suspensions were designed, and each concentration had three replicates, where each replicate contained one gram of live *Artemia* (body length of 4–5 mm). After soaking for 30 min, the APS content inside the live *Artemia* was measured using UV spectrophotometry (UV-2700, Shimadzu, Kyoto, Japan) [29]. The results showed that the APS contents inside the *Artemia* corresponding to 0, 2, 4, 8, and 16% APS suspensions were 0.114 ± 0.014, 0.176 ± 0.017, 0.241 ± 0.024, 0.229 ± 0.014, and 0.226 ± 0.012 g/g, respectively. Statistical analysis showed that there was no significant difference among 4, 8, and 16%, but all three were significantly higher than 0% and 2%. Therefore, in the present study, live *Artemia* (body length of 4–5 mm) soaking in the 4% APS suspension for 30 min was adopted as the administration protocol of APSs.

### 2.3. Experiment 1: Effects of APSs on the Survival Rate, Growth, and Immunity of Seahorse Juveniles During Diet Conversion

#### 2.3.1. Experiment 1 Protocol

The experimental seahorses (with a body height of approximately 7.5 cm) were divided into two groups, namely the APS group and the control group. Each group consisted of three replicates, and each replicate contained 50 seahorses and placed in an independent rearing tank (0.7 m in diameter and 0.8 m in depth). The seahorses in two groups were both fed with live *Artemia* at 7:00 a.m., followed by frozen *Mysis* at 8:30 a.m. and 3:00 p.m. every day. In contrast, seahorses in the APS group were fed with *Artemia* that was soaked in 4% APS suspension for 30 min, and seahorses in the control group were fed with *Artemia* that was not soaked in APS suspension. The feces and uneaten frozen shrimp were siphoned out two hours after each feeding. The rearing tanks were continuously flowed with fresh seawater for 12 h a day at a flowrate of 1.2–1.5 L/min. The parameters of seawater were a temperature of 26–27 °C, salinity of 30–31‰, dissolved oxygen content of 6.0–6.5 mg/L, and pH of 8.0–8.2, respectively. The experiment lasted for 30 days.

#### 2.3.2. Survival Rate and Growth Measurement

During the experiment, the dead seahorses (if any) from each replicate of each group were timely picked out every day and recorded for calculating the final survival rate. On the last day of the experiment, 20 surviving seahorses from each replicate of each group were taken for measuring their body height and wet body weight. Body height was measured with a ruler with a precision of 0.1 cm (i.e., 1 mm), and the wet body weight was measured using a balance (XPR303S/AC, Mettler Toledo, Zurich, Switzerland) with a precision of 0.001 g (i.e., 1 mg).

#### 2.3.3. Plasma Immunocytokine Determination

On the last day of the experiment, six surviving seahorses were randomly sampled from each replicate of each group for blood collection. Each seahorse was first anesthetized with a solution of MS-222 (50 mg/L) (Sigma-Aldrich, St. Louis, MO, USA) in seawater, and then a quarter of the tail was cut off. The remaining tail of the amputated seahorse was immediately inserted into a 2-mL centrifuge tube containing 0.8 mL of anticoagulant (citric acid 0.48 g, sodium citrate 1.32 g, glucose 1.47 g, and distilled water 100 mL) and dipped into the anticoagulant. Blood spontaneously flowed out of the caudal artery and mixed with the anticoagulant. Due to the small volume of blood in each seahorse, the blood from each of the three seahorses was pooled in the same centrifuge tube. The mixture of blood and anticoagulant was left to stand for 10 min at 4 °C and then centrifuged at 840× *g* for 10 min at 4 °C (Sigma 3K18, Sigma-Aldrich, St. Louis, MO, USA) to collect the supernatant (i.e., plasma). The plasma was stored at −80 °C until use for the determinations of Ig M, IL-2, IFN-α, and plasma protein. Ig M, IL-2, IFN-α, and plasma protein were determined with commercial ELISA kits (Nanjing Jiancheng Bioengineering Institute, Nanjing, China) for fish plasma following the manufacturer’s instructions. The units of Ig M, IL-2, and IFN-α were expressed as the amount contained in each milligram of plasma protein.

#### 2.3.4. Intestinal Microbiota Analysis

After blood collection, the surface of the seahorse was disinfected with 75% (*v*/*v*) of alcohol, then placed on an ultra-clean workbench (BBS-H1500, BioBase, Shandong, China) and dissected aseptically for intestinal tract collection. The intestinal tracts of each of the three seahorses were pooled in the same frozen tube (Rnase/Dnase-free) and stored at −80 °C until use for the analysis of the intestinal microbiota composition. This analysis was conducted with 16S rDNA sequencing [30]. Six indices, including Chaol richness estimator (Chao1), observed species richness (Observed species), Simpson diversity index (Simpson), Shannon Wiener’s diversity index (Shannon), Pielou’s evenness index (Pielou’s-e), and Good’s nonparametric coverage estimator (Good’s coverage), were used to assess the alpha diversity of the intestinal microbiota.

### 2.4. Experiment 2: Effects of APSs on the Reproductive Performance of Seahorse Broodstocks

#### 2.4.1. Experiment 2 Protocol

The experimental seahorses (with a body height of approximately 11 cm) were divided into two groups, namely the APS group and the control group. The seahorses in each group were separated by gender and reared separately. The seahorses in each group were fed with live *Artemia* at 7:00 a.m., followed by frozen *Mysis* at 8:30 a.m. and 3:00 p.m. every day. In contrast, seahorses in the APS group were fed with APSs-soaked *Artemia*, while seahorses in the control group were fed with APSs-unsoaked *Artemia*. The daily management and rearing conditions were the same as in Experiment 1. After one month of feeding, six female seahorses were randomly taken from each group to collect their ovaries for metabolite component analysis, and another 20 males and 20 females were selected from each group to pair them in one male and one female, for a total of 20 pairs, for reproductive performance observation. Each pair was placed in an independent breeding tank (0.8 m in diameter and 1.0 m in depth), and the identities of these pairs were named after the tank numbers they were in, namely Tank #1 to Tank #20. During the pairing period, seahorses stopped feeding on live *Artemia* but continued to feed on frozen *Mysis* twice a day.

#### 2.4.2. Ovarian Metabolite Component Analysis

The female seahorses were first anesthetized with a solution of MS-222 (50 mg/L) in seawater and then dissected to collect their ovaries for metabolite component analysis. Metabolite components were determined using ultra-high performance liquid chromatography-tandem mass spectrometry (UHPLC-MS/MS). Briefly, 60 mg of the ovary samples were homogenized in 200 μL of distilled water. Then, the metabolites were extracted with 800 μL of methanol:acetonitrile solution (1:1, *v*/*v*) and vacuum freeze-dried. The extract was dissolved in 100 μL of acetonitrile:water (1:1, *v*/*v*), and 2 μL of this solution was taken to add onto an ACQUITY UPLC BEH Amide column (1.7 μm, 2.1 mm × 100 mm) (Waters Technologies Ireland Ltd., Wexford, Ireland) for hydrophilic interaction liquid chromatography with an Agilent 1290 Infinity UHPLC system (Agilent Technologies, Santa Clara, CA, USA) [31].

#### 2.4.3. Mating Success Rate, Assessment Time Required Before Mating, Brood Size, and Newborn Body Height

After pairing, the mating status of each pair was observed and checked daily for two weeks. The criterion for mating success was to simultaneously observe an obvious expansion of the male’s pouch (due to the reception of eggs) and an obvious contraction of the female’s abdomen (due to the expulsion of eggs). Once mating success was observed, the identity and mating date of the pair were recorded for calculating the mating success rate and the assessment time required before mating. After giving birth, its number of newborns was counted, and the body height of ten randomly-sampled newborns was measured. The mating success rate refers to the percentage of successfully-mated pairs among the 20 pairs, while the assessment time required before mating refers to the time from the day of pairing to the day of successful mating. Brood size refers to the total number of newborns in a brood. Body height, a key indicator to evaluate the quality of the newborn [32], refers to the straight-line distance from the tip of the head coronet to the tip of the uncurled tail.

### 2.5. Statistical Analysis

All data were expressed as the mean ± standard deviation (SD) and were analyzed using Origin PRO (v9.8.0.200) graphing and analysis software. The differences between the control and APS groups in both two experiments were analyzed using the independent sample *t*-test. The *p* value of 0.05 was taken as a significance level. Before running the *t*-test, the normality of all data was tested using the Shapiro–Wilk test.

## 3. Results

### 3.1. Effects of APSs on the Survival Rate, Growth, and Immunity of Seahorse Juveniles During Diet Conversion

#### 3.1.1. Survival Rate and Growth

**Figure 1:**
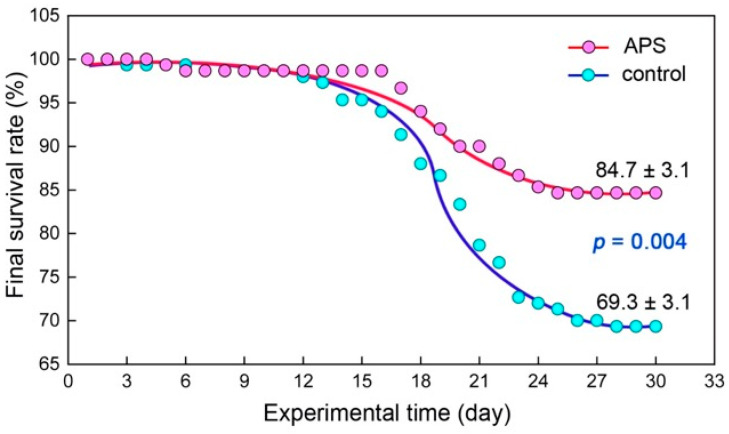
Final survival rates of seahorse juveniles in the control group and the APS group in Experiment 1.

**Figure 2:**
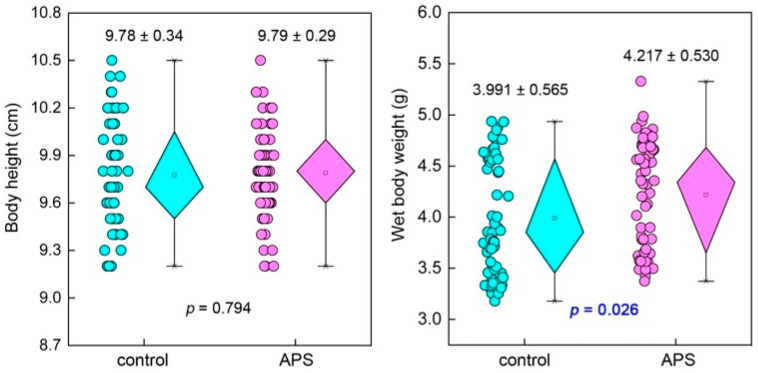
The body height and wet body weight of seahorse juveniles in the control group and the APS group in Experiment 1.

Throughout the entire experiment, both groups of seahorses died to varying degrees, and most of the deaths occurred between the 15th and 24th days of the experiment. However, the number of dead individuals in the APS group was obviously less than that in the control group. The final survival rates of the APS group and the control group were 84.7 ± 3.1% and 69.3 ± 3.1%, respectively, with a significant difference between the two (*p* = 0.004) (Figure 1). As for the growth, the body height of the APS group and the control group both reached about 9.8 cm at the end of the experiment, and there was no significant difference between them (*p* = 0.794). However, in terms of the wet body weight, the level of the APS group (4.217 ± 0.530 g) was significantly heavier than that of the control group (3.991 ± 0.565 g) (*p* = 0.026) (Figure 2).

**Figure 3:**
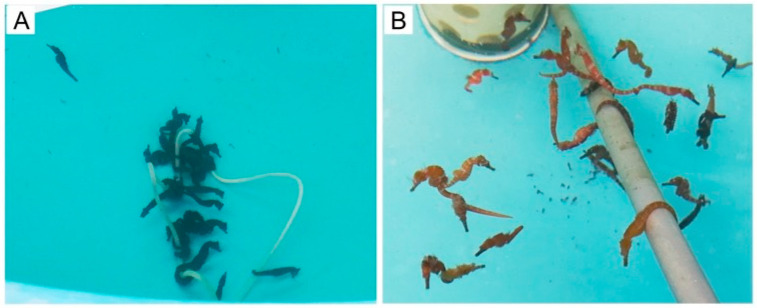
The body color of seahorse juveniles in the control group (**A**) and the APS group (**B**) in the later stage of Experiment 1.

In addition, at the later stage of the experiment, the body color of the seahorses in the APS group was also different from that in the control group. Specifically, the seahorses in the APS group often appeared reddish brown in the morning and dusk (Figure 3B) while the seahorses in the control group were always black or gray black (Figure 3A).

#### 3.1.2. Plasma Immunocytokines

**Figure 4:**
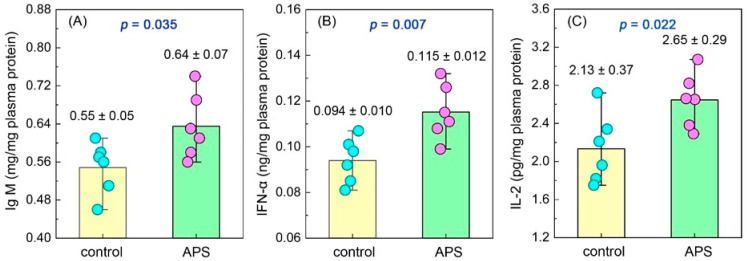
The contents of the plasma Ig M (**A**), IFN-α (**B**) and IL-2 (**C**) of seahorse juveniles in the control group and the APS group in Experiment 1.

At the end of the experiment, the plasma Ig M in the seahorses of the APS group was 0.64 ± 0.07 mg/mg plasma protein, which was significantly higher than that of the control group (0.55 ± 0.05 mg/mg plasma protein) (*p* = 0.035) (Figure 4A). Similarly, the plasma IFN-α (0.115 ± 0.012 vs. 0.094 ± 0.010 ng/mg plasma protein, *p* = 0.007) and plasma IL-2 (2.65 ± 0.29 vs. 2.13 ± 0.37 pg/mg plasma protein, *p* = 0.022) were also significantly higher in the APS group than in the control group (Figure 4B,C).

#### 3.1.3. Intestinal Microbiota

**Figure 5:**
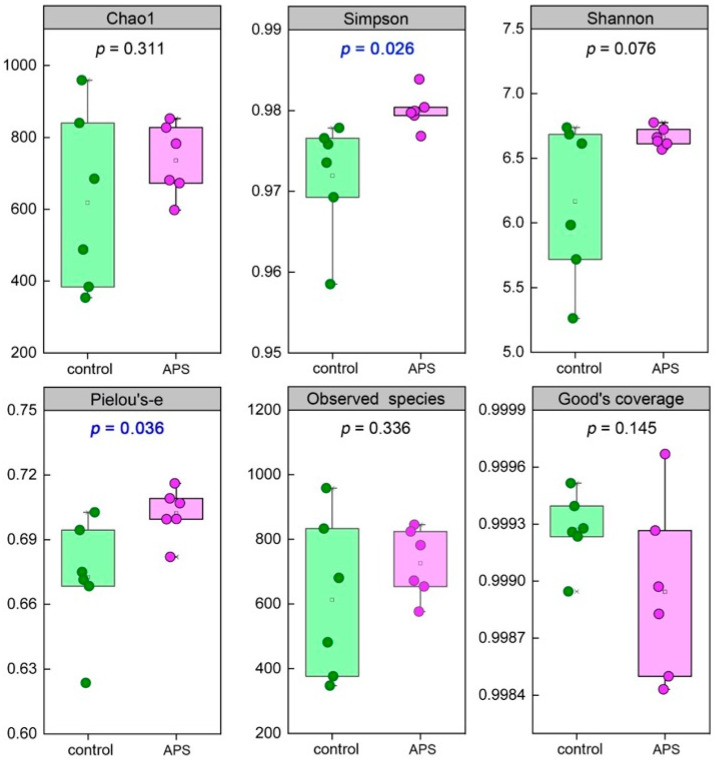
The alpha diversity of intestinal microbiota in seahorse juveniles of the control group and the APS group in Experiment 1.

**Figure 6:**
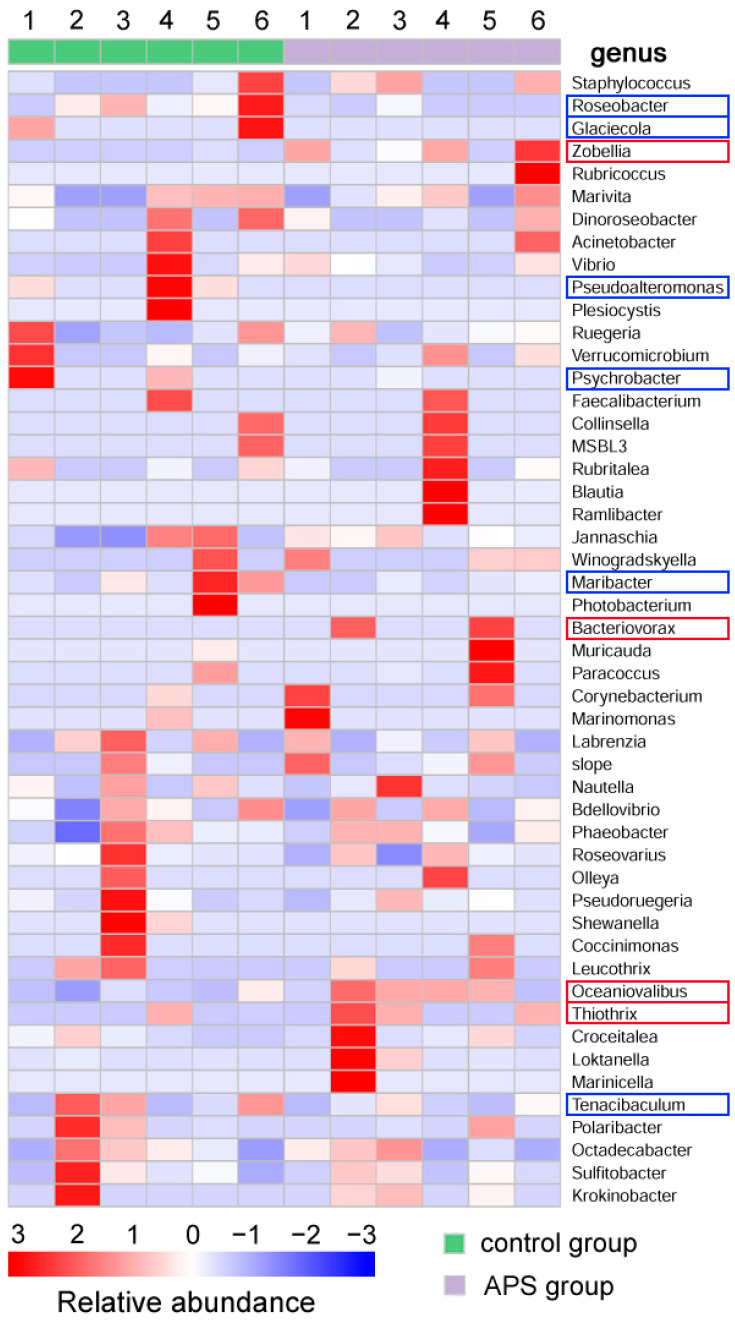
The composition of intestinal microbiota in seahorse juveniles of the control group and the APS group in Experiment 1. The intestinal microbiota listed in the heatmap are the top 50 genera in terms of average abundance. The bacterial genera with red borders are those with higher abundance in the APS group than in the control group, while the bacterial genera with blue borders are those with higher abundance in the control group than in the APS group.

As for the alpha diversity of intestinal microbiota, two indices, the Simpson (indicating microbial community diversity) (*p* = 0.026) and Pielou’s-e (indicating microbial community evenness) (*p* = 0.036) of the APS group were significantly higher than those of the control group, while the other four indices had no significant difference (Figure 5). Among the top 50 intestinal microbiota with average abundance ranking, the abundance of genera of *Zobellia* (mean value: 0.0139 vs. 0.0004), *Bacteriovorax* (0.0023 vs. 0), *Oceaniovalibus* (0.0368 vs. 0.0122), and *Thiothrix* (0.0017 vs. 0.0005) in the APS group was higher than that in the control group (Figure 6, red border), while the abundance of genera of *Roseobacter* (0.0054 vs. 0.0005), *Glaciecola* (0.0012 vs. 0), *Pseudoalteromonas* (0.0038 vs. 0), *Psychrobacter* (0.0063 vs. 0.0004), *Maribacter* (0.0168 vs. 0.0030), and *Tenacibaculum* (0.0065 vs. 0.0027) in the control group were higher than those in the APS group (Figure 6, blue border).

### 3.2. Effects of APSs on the Reproductive Performance of Seahorse Broodstocks

#### 3.2.1. Ovarian Metabolite Components

**Figure 7:**
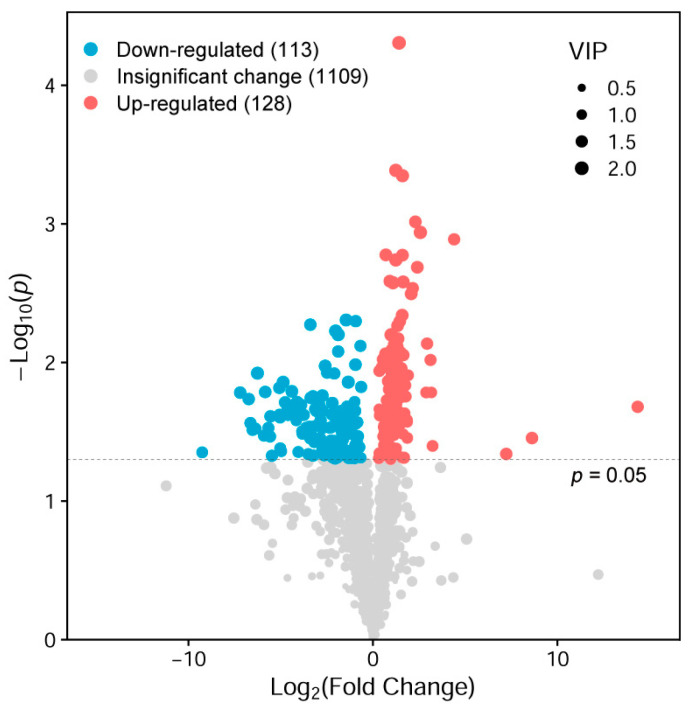
Volcano plot of significantly differential metabolites (SDMs) in the ovaries of seahorse broodstocks between the control group and the APS group in Experiment 2. Using fold change (FC) ≥1.5 as the baseline, the red plots indicate the abundance-upregulated SDMs in the APS group compared with the control group, and the blue plots indicate the abundance-downregulated SDMs in the APS group compared with the control group. The size of the plot indicates the VIP (variable importance in the projection) value of the OPLS-DA model. The dashed line is the critical line for *p* = 0.05.

**Figure 8:**
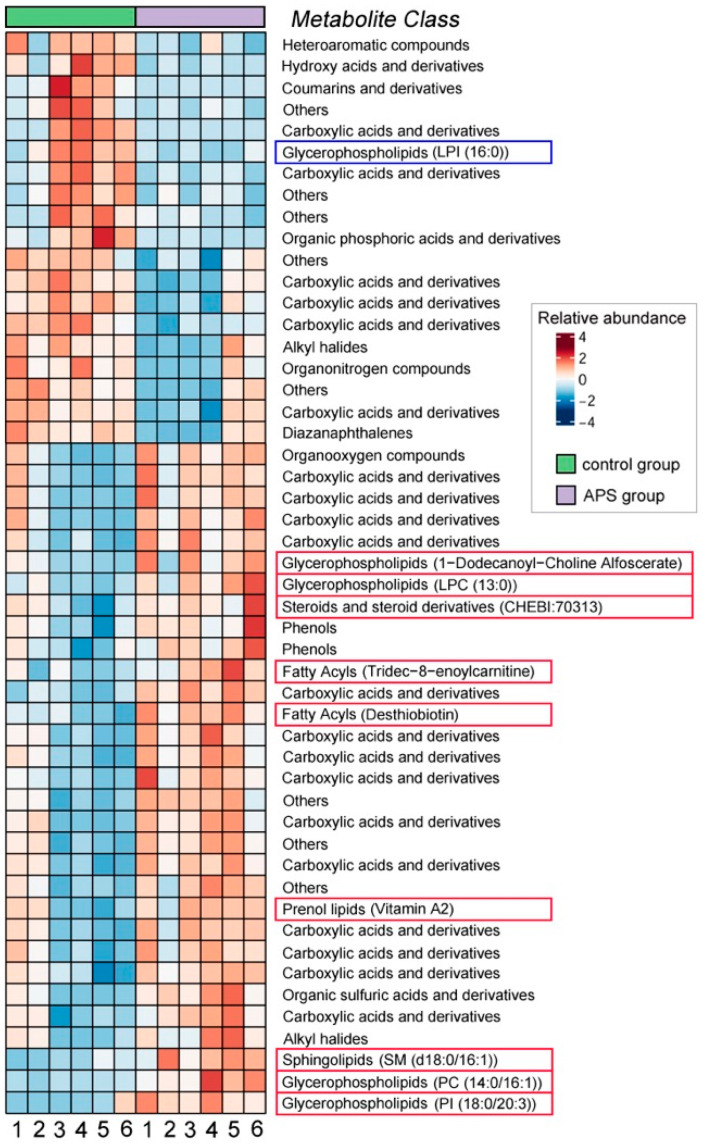
The top 50 significantly differential metabolites (SDMs) in VIP (variable importance in the projection) values in the ovaries of seahorse broodstocks between the control group and the APS group in Experiment 2. The SDMs with red borders are those lipids whose abundance was upregulated in the APS group, and the SDMs with blue borders were those lipids whose abundance was downregulated in the APS group.

The metabolomic data showed that there were 141 significantly differential metabolites (SDMs) in the ovaries between the APS group and the control group. Taking the control group as the baseline, the relative abundance of 128 SDMs in the APS group was upregulated, while the relative abundance of 113 SDMs was downregulated (Figure 7). Among the top 50 SDMs in VIP (variable importance in the projection) values, metabolites with upregulated relative abundance in the APS group included many lipids, such as glycerophospholipids (1-dodecanoyl-choline alfoscerate, LPC (13:0), PC (14:0/16:1) and PI (18:0/20:3)), steroids and steroid derivatives (CHEBI: 70313), prenol lipids (vitamin A2), sphingolipids (SM (d18:0/16:1)), and fatty acyls (tridec-8-enoylcarnitine and desthiobiotin) (Figure 8, red border), indicating that the lipids in the ovaries of the APS group were more abundant than those of the control group.

#### 3.2.2. Mating Success Rate and Assessment Time Required Before Mating

The mating success rate of the APS group was 65% (13/20), and that of the control group was 60% (12/20). The assessment time required before mating of the successfully-mated pairs in the APS group was 4.92 ± 1.55 day, which was significantly shorter than 7.92 ± 1.38 day in the control group (*p* < 0.001) (Table 1).

#### 3.2.3. Brood Size and Newborn Body Height

**Figure 9:**
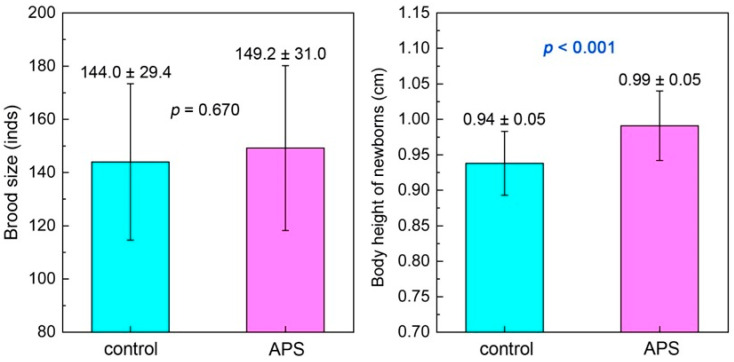
The brood size and the newborn body height of seahorse broodstocks in the control group and the APS group in Experiment 2.

The brood size of the APS group was 149.2 ± 31.0 inds, with no significant difference compared with the 144.0 ± 29.4 inds of the control group (*p* = 0.670). For the newborn body height, the value of the APS group was 0.99 ± 0.05 cm, which was significantly higher than 0.94 ± 0.05 cm of the control group (*p* < 0.001) (Figure 9).

## 4. Discussion

The results of the present study showed that dietary APS could significantly improve the growth, survivorship, plasma immune levels, and intestinal microbiota diversity of seahorse juveniles during diet conversion as well as the ovarian lipid content, mating assessment time, and newborn body height of seahorse broodstocks during breeding. These results indicate that the promoting effects of APSs on growth, immune function, antibacterial activity, and intestinal homeostasis are also applicable to the lined seahorse (*H. erectus*), just like other aquaculture animals. Conspicuously, these results also indicate that APSs have the effect of improving reproductive performance, which has rarely been reported in other aquaculture animals. Taken together, in the present study, we have demonstrated not only the applicability of APSs in the seahorse, but also the feasibility of using live *Artemia* as the carrier for APS administration.

### 4.1. APSs Improve the Immune Capacity of Seahorse Juveniles

One of the most prominent effects of APSs is immune improvement. Many applications have shown that it can improve the phagocytic ability and respiratory burst activity in the blood cells of tilapia (*Oreochromis niloticus*) [33], induce the mRNA expression of interleukin-1β in the head kidney of common carp (*Cyprinus carpio* L.) [34] and toll-like receptors in the liver of turbot (*Scophthalmus maximus* L.) [19], activate the pro-phenoloxidase and increase the immunocytokine content in the plasma of giant freshwater prawn (*Macrobrachium rosenbergii*) [17], and enhance the disease resistance of catla (*Catla catla*) [35], Asian seabass (*Lates calcarifer*) [16], and crucian carp (*Carassius auratus*) [20]. In recent years, APSs, as an immunostimulant, have been increasingly applied in aquaculture.

Ig M, IL-2, and IFN-α are three immunocytokines closely related to immune status. Their high levels usually mean that the fish has a relatively good immune status [24,25]. In the present study, after dietary APSs, the Ig M, IL-2, and IFN-α in the plasma of the seahorse increased, which intuitively indicated that the immune capacity of the seahorse was stimulated by APSs. In other words, the immune-promoting effect of APSs was also workable in the seahorse. In addition to the direct evidence of plasma immunocytokines, there were two indirect pieces of evidence that could also indicate APSs improving the immune status of the seahorse. The first was the survival rate. The immune enhancement stimulated by APSs largely synergistically enhanced the bacterial resistance, free-radical scavenging activity, and pathogen killing ability of the seahorse, thereby leading to a significant increase in survival rate. The second was body color. Seahorses are a kind of ornamental fish and can present a variety of beautiful body colors (e.g., bright yellow, orange, reddish brown, and light green). The presentation of beautiful body color usually means that the seahorse is in a good physical condition, because color presentation is a high-energy consumption behavior, and usually only healthy individuals can afford this behavior [36,37,38]. In our previous study, we observed that the seahorses fed live *Mysis* exhibited a brighter body color compared with the seahorses fed frozen *Mysis* [39], and in the present study, the body color of seahorses treated with APSs was brighter than that of the untreated seahorse, both suggesting that the immune-health status of the seahorses had been greatly improved to the extent that they could afford the behavior of color presentation.

As for the mechanism of APSs promoting the immunity of aquatic animals, many researchers have also carried out extensive studies [40,41]. After APSs are ingested and transferred to the intestinal tract, some intestinal bacteria are induced, and they break down the polysaccharides into fermentable monosaccharides by encoding numerous carbohydrate-active enzymes (CAZymes) [42]. These fermentable monosaccharides are further broken down by other microorganisms to form various bioactive metabolites. Among these metabolites, there is a type that can stimulate the proliferation of macrophages and lymphocytes, probably through the activation of the TLR4-MyD88-dependent pathway through TLR4, which subsequently activates key nodes in the pathway (e.g., TRAF-6 and NF-κB) [43]. Macrophages are essential immune cells that participate in stimulating innate immunity, which are also crucial for the adaptive immunological response. Depending on different external stimuli, macrophages are activated and differentiated into M1 or M2 macrophages. M1 macrophages can produce proinflammatory cytokines and synthesize inducible nitric oxide synthase, mainly participating in the elimination of pathogens and the initiation of inflammation [44,45]. Lymphocytes can produce various immune cytokines, including antibodies, interleukins, and interferons, through different cellular subsets, promoting the growth and activation of other immune cells, thereby killing and clearing intracellular pathogenic infections [46]. The immune mechanism of APSs provides a good explanation for why the content of plasma immunocytokines (i.e., Ig M, IL-2, and IFN-α) in the APS group increased in the present study.

### 4.2. APSs Improve the Growth of Seahorse Juveniles

In addition to the effect of immune-promoting, another prominent effect of APSs, namely growth-promoting, was also verified in the present study. We found that the body height of the APS group was not different from that of the control group, but the body weight was significantly heavier than that of the control group. The reason why there was no difference in body height but a difference in body weight may be that seahorse juveniles with a body height of approximately 9.5 cm begin to prepare for reproduction, and so they allocated more energy to their body weight for the development of gonads and brood pouches, resulting in a reduction in the energy allocated to body height [8]. As for the reason why APSs can promote growth, some studies believe that this is related to the fact that APSs have the property of improvement in digestive enzyme activities, digestive juice secretion, and absorption of nutrients [15,47].

### 4.3. APSs Improve the Intestinal Microbiota of Seahorse Juveniles

The homeostasis of intestinal microbiota has a profound impact on host health. The high richness, evenness, and diversity of the intestinal microbiota usually mean that the host has good intestinal homeostasis, which in turn represents that the host is in a healthy status [48,49]. In the present study, after dietary APSs, two alpha diversity indices of intestinal microbiota, the Simpson (indicating microbial community diversity) and Pielou’s-e (indicating microbial community evenness), were significantly increased, which indicated that the health status of the seahorses had been significantly improved. As for the mechanism of APSs improving intestinal homeostasis, some researchers have also conducted relevant studies. Polysaccharides, acting as carbon sources, are involved in regulating the makeup, development, metabolism, biological activity, and metabolite synthesis of intestinal microbiota [42]. Moreover, polysaccharides can also be degraded by intestinal microbiota (especially *Bacteroidetes*, *Firmicutes*, and *Actinobacteria*) into prebiotics that can be absorbed by the host and are highly beneficial to the host’s health [41,50].

### 4.4. APSs Improve the Reproductive Performance of Seahorse Broodstocks

Although the effects of APSs in promoting immunity and growth have been widely demonstrated in fish [15,16,17,18], its effect in promoting reproduction has rarely been reported. In the present study, we found that APSs also had the effect of promoting reproduction, which was specifically shown as follows: compared with the control group, the assessment time required before mating for male and female seahorses in the APS group was significantly shortened, and the body height of the newborns was significantly increased. This improvement in reproductive performance may mainly be attributed to the immune enhancement induced by APSs, that is, the improvement in reproductive performance is also one of the intuitive manifestations of seahorse immune enhancement. In our previous study, we found that immunity and health status are important criteria for seahorse mate choice. Individuals with strong immunity usually show a beautiful body color and active courtship behavior, and they spend significantly less time in assessing their mates and succeeding in mating than individuals with poor body color and inactive courtship behavior [39]. In addition, seahorses are monogamous species [51], and they rarely change their mates during their life, but once their mates have health problems, they will switch mates [52]. This fact also illustrates the importance of immune capacity in seahorse mate choice.

Moreover, it may be precisely due to the immune enhancement induced by APSs that the energy investment for seahorses to maintain their own immunity can be appropriately reduced, thereby allocating more energy to other aspects such as reproduction (e.g., lipid synthesis in ovaries). In the present study, we found that the expression abundance of lipids (e.g., glycerophospholipids, steroids, prenol lipids, sphingolipids, and fatty acyls) in the ovary of the APS group was significantly higher than those of the control group. Lipids in the ovaries or eggs are the most important substances for embryonic development. They not only provide the primary energy for developing embryos, but are also involved in many biological functions such as embryonic pigmentation, organogenesis (e.g., eyes and brain) and neurological development, embryonic immune regulation, and lipid metabolism regulation [53,54]. Eggs with high lipid content are widely regarded as high-quality eggs [27]. In the present study, it is precisely because the ovaries in the APS group contained more abundant lipids that their embryos developed better, and their newborns’ body height was significantly higher than that in the control group.

## 5. Conclusions

In the present study, we used live *Artemia* as the carrier of APSs to feed the seahorses (*H. erectus*). The results showed that APSs could not only improve the survival rate, growth, and immune capacity of seahorse juveniles during diet conversion, but also improve the reproductive performance of seahorse broodstocks. These results provide guidance for the healthy cultivation of seahorses and the administration route of APSs in other cultured fish.

## Figures and Tables

**Table 1:** The mating status and the assessment time required before the mating of seahorses in the control group and the APS group in Experiment 2.

Tank No.	Control		APS	
	Mating Status	^1^ Time (Day)	Mating Status	Time (Day)
1#	√	6	−	
2#	√	7	√	6
3#	−		√	5
4#	−		√	3
5#	√	8	−	
6#	√	6	√	7
7#	−		−	
8#	√	10	√	6
9#	√	7	√	5
10#	√	9	√	3
11#	−		−	
12#	−		√	3
13#	√	7	√	5
14#	√	8	√	4
15#	√	8	−	
16#	−		√	8
17#	√	9	√	4
18#	−		−	
19#	√	10	−	
20#	−		√	5
Total	12		13	
Mean		7.92 ± 1.38	(*p* < 0.001)	4.92 ± 1.55 *

^1^ Time: the assessment time required before mating (i.e., the time from the day of pairing to the day of successful mating). “√”: Successfully-mated pair; “−”: Pair without successful mating within the two-week observation period. “*” indicates a significant difference between the control group and the APS group.

## Data Availability

The original data presented in the study are included in the article; further inquiries can be directed to the corresponding author.
